# PAIN DURING MIDLIFE: A CROSS-NATIONAL ANALYSIS OF COHORT DIFFERENCES IN PAIN IN THE US, EUROPE, SOUTH KOREA, AND MEXICO

**DOI:** 10.1093/geroni/igae098.2197

**Published:** 2024-12-31

**Authors:** Frank Infurna, Orchee Syed, Yesenia Cruz-Carrillo, Nutifafa Dey, Markus Wettstein, Kevin Grimm, Margie Lachman, Denis Gerstorf

**Affiliations:** Arizona State University, Tempe, Arizona, United States; Arizona State University, Tempe, Arizona, United States; Arizona State University, Tempe, Arizona, United States; Arizona State University, Tempe, Arizona, United States; Humboldt University Berlin, Berlin, Berlin, Germany; Arizona State University, Tempe, Arizona, United States; Brandeis University, Waltham, Massachusetts, United States; Humboldt University Berlin, Berlin, Berlin, Germany

## Abstract

Over the past several decades, middle-aged Americans have exhibited troubling trends of declining mental and physical health over successive cohorts. However, this trend has not been consistently observed in peer nations. It is an open question whether pain shows similar national disparities. We thus examine how self-reports of pain have historically changed in the U.S. during midlife, and how this compares to peer nations. We used harmonized data on pain from nationally representative longitudinal panel surveys from the U.S., 13 European nations, South Korea, and Mexico to directly quantify national similarities and differences in historical change in midlife pain. Our results corroborate earlier reports that midlife pain is higher amongst later-born cohorts in the U.S. Of note is that these historical increases emerged at earlier stages of midlife for later-born cohorts. A similar pattern of historical increases in pain was observed in Continental and Nordic Europe. In contrast, historical declines in pain were observed in England, Mediterranean Europe, South Korea, and Mexico. These results suggest there could be aspects of American midlife today that are exacerbating feelings of pain, and these aspects may be shared in some European nations but absent or less influential in other peer nations. Our discussion focuses on potential explanations for this pattern, such as population level discrepancies in health, differential use of health care services, and the inter/intrapersonal costs of westernization, as well as how pain is conceptualized across nations.

